# Analytical Exploration of Potential Pathways by which Diabetes Mellitus Impacts Tuberculosis Epidemiology

**DOI:** 10.1038/s41598-019-44916-7

**Published:** 2019-06-11

**Authors:** Susanne F. Awad, Soha R. Dargham, Ryosuke Omori, Fiona Pearson, Julia A. Critchley, Laith J. Abu-Raddad

**Affiliations:** 1 Infectious Disease Epidemiology Group, Weill Cornell Medicine-Qatar, Cornell University, Qatar Foundation, Education City, Doha, Qatar; 2 Population Health Research Institute, St George’s, University of London, London, UK; 3 Division of Bioinformatics, Research Center for Zoonosis Control, Hokkaido University, Sapporo Hokkaido, Japan; 4 Japan Science and Technology Agency, PRESTO, Kawaguchi Saitama, Japan; 5 Department of Healthcare Policy and Research, Weill Cornell Medicine, Cornell University, New York, New York, USA; 6 College of Health and Life Sciences, Hamad bin Khalifa University, Qatar Foundation, Education City, Doha, Qatar

**Keywords:** Diabetes, Bacterial infection

## Abstract

We aimed to develop a conceptual framework of diabetes mellitus (DM) effects on tuberculosis (TB) natural history and treatment outcomes, and to assess the impact of these effects on TB-transmission dynamics. The model was calibrated using TB data for India. A conceptual framework was developed based on a literature review, and then translated into a mathematical model to assess the impact of the DM-on-TB effects. The impact was analyzed using TB-disease incidence hazard ratio (HR) and population attributable fraction (*PAF*) measures. Evidence was identified for 10 plausible DM-on-TB effects. Assuming a flat change of 300% (meaning an effect size of 3.0) for each DM-on-TB effect, the HR ranged between 1.0 (*Effect 9-Recovery*) and 2.7 (*Effect 2-Fast progression*); most effects did not have an impact on the HR. Meanwhile, TB-disease incidence attributed directly and indirectly to each effect ranged between −4.6% (*Effect 7-TB mortality*) and 34.5% (*Effect 2-Fast progression*). The second largest impact was for *Effect 6-Disease infectiousness* at 29.9%. In conclusion, DM can affect TB-transmission dynamics in multiple ways, most of which are poorly characterized and difficult to assess in epidemiologic studies. The indirect (e.g. onward transmission) impacts of some DM-on-TB effects are comparable in scale to the direct impacts. While the impact of several effects on the HR was limited, the impact on the *PAF* was substantial suggesting that DM could be impacting TB epidemiology to a larger extent than previously thought.

## Introduction

Tuberculosis (TB) disease burden remains high in parts of the world^1,2^. A quarter of the world’s population has been infected with *M*. *tuberculosis*, of whom a fraction will develop active disease within their lifetime^1,2^. In 2017, 10.0 million incident cases were estimated with TB disease and 1.3 million died from it^1,2^. There is a recognition that major reduction in TB burden is difficult to achieve without controlling its risk factors. The World Health Organization’s (WHO) post-2015 TB strategy calls for prioritization of interventions addressing the key TB risk factors including diabetes mellitus (DM)^3^.

A synergetic relationship between TB and DM has been suspected for decades^4^, but has only recently emerged as a global-health concern, with the growing DM prevalence in TB endemic regions^5,6^, Globally, an estimated 425 million people live with DM; a number that is expected to grow to 629 million by 2045^7^. Low- and middle-income countries are the epicenter of the increasing DM burden accounting for over 80% of global DM cases^7^.

DM appears to increase the risk of TB disease by about three-fold^5,6,8^, and to have profound adverse impact on TB-treatment outcomes (e.g. DM appears to increase the risk of TB death by two to four-fold, and TB disease relapse and recurrence by two-fold, among others)^6,9–13^. DM is suspected to account for a considerable proportion of TB-disease incident cases^14–21^, highlighting the importance of the joint TB-DM epidemic. Yet, our understanding of the underlying biological/epidemiological interactions between TB and DM remains limited. It is critical to delineate these complex interactions to assess both the direct and indirect implications of DM on TB’s burden^6,22^.

Most indirect implications of TB-DM interactions relate to onward transmission of TB infection. DM may increase the risk of development of TB disease, which is a *direct* (etiological) effect for DM on TB. However, with the ensuing pool of infectious individuals with TB disease, TB transmission would increase leading to more individuals with TB infection. The latter onward-transmission effect is an *indirect* effect of DM increasing the risk of development of TB disease. While conventional population attributable fraction (*PAF*) approaches (such as *Levin’s formula*^23^) can estimate the direct population impact of DM on TB disease, they do not account for the indirect impacts. The latter, however, can be captured and estimated through mathematical modeling of TB-transmission dynamics in the population.

**Figure 1:**
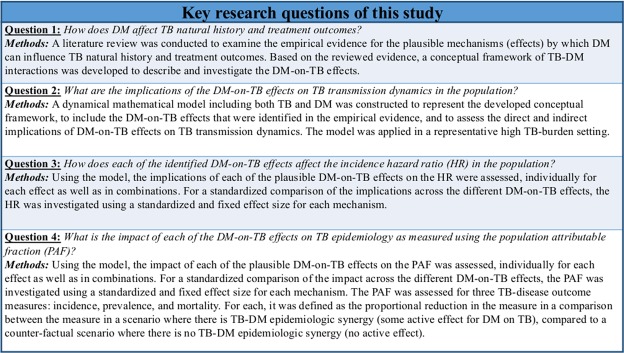
List of the key research questions assessed in this study along with the methods used to address them.

Against this background, we aimed first to develop a conceptual framework that describes the different possible pathways by which DM could affect TB natural history and treatment outcomes. Second, we translated this conceptual framework into a population-based mathematical TB-DM model incorporating these effects and their *direct* and *indirect* impacts. Third, we assessed the impacts of these effects (using a “flat change” of 300% to standardize the effect size) on TB epidemiology by applying the model in a representative high TB-burden setting. The study thus provides a theoretical analytical investigation of the implications of the DM effects (alone and in combinations) on TB epidemiology for an improved understanding of the TB-DM synergy. Using a standardized effect size also enabled the investigation and comparison of the theoretical importance of both direct and indirect impacts for each potential pathway. The research questions along with the corresponding methods are summarized in Figure 1. This study approach was deemed necessary as we do not yet have sufficient empirical evidence to quantify the precise and exact effect sizes of most of these DM-on-TB effects. Therefore, our work presents an original and comprehensive theoretical assessment of the potential TB-DM interactions and their implications on TB epidemiology, particularly how important indirect (or true population) effects might be for each pathway. It does not aim to provide precise information on the absolute actual impact of each pathway on TB epidemiology.

## Methods

### Conceptual framework for TB-DM interactions

We conducted a literature review based on which we developed a conceptual framework of TB-DM interactions. Available publications on the TB-DM interactions were reviewed through searches using the PubMed and Google Scholar databases up to May 2017. For inclusiveness, we used broad search criteria with terms exploded to cover all subheadings. Any publication reporting on TB-DM interactions qualified for inclusion in this review. No language or year restrictions were imposed. The review identified the plausible mechanisms and effects by which DM can influence TB’s natural history and treatment outcomes.

### Mathematical model, data sources, and model fitting

We constructed a population-based dynamical TB-DM mathematical model to represent the developed conceptual framework and assess the direct and indirect impacts of the effects of DM on TB. The model is an adaptation of an earlier TB-transmission model^24^ that was extended to include DM and the postulated TB-DM interactions. The model consisted of a system of coupled nonlinear differential equations stratifying the population according to TB infection status and stage, disease form, treatment status, and recovery status.

**Figure 2:**
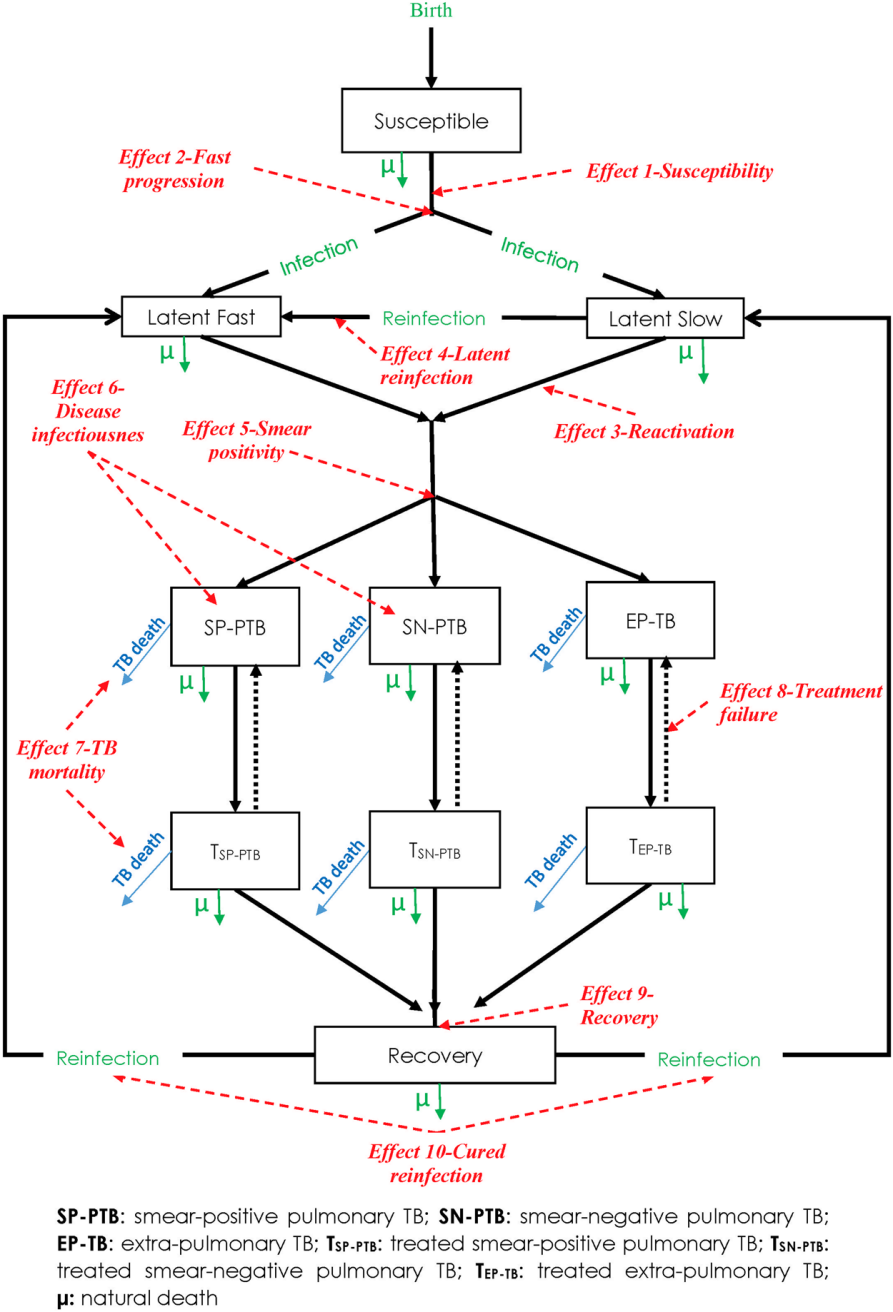
A conceptual framework for the TB-DM epidemiologic synergy. The black lines indicate the transitions within TB’s natural history and treatment states. The red lines indicate the 10 plausible effects of DM on TB natural history and treatment outcomes.

The model incorporated two TB natural histories depending on DM status (Fig. 2). DM was parsimoniously included in the model by stratifying a specific and fixed proportion of the population to be living with DM. DM-free individuals were also assumed to be susceptible but at risk of TB infection. Newly infected individuals progress into either latent-slow TB infection (LSI) or latent-fast TB infection (LFI). Individuals with LSI develop TB disease at a rate corresponding to a 5% lifetime risk of developing TB disease^24–27^—meaning that the risk of developing TB disease is so small that only 5% of individuals in LSI will eventually develop the disease, simply because they would have reached the end of their life expectancy before developing TB disease. Individuals with LFI develop TB disease within a short duration after infection^24–26^.

TB disease was stratified into three clinically-relevant forms: smear-positive pulmonary (SP-PTB), smear-negative pulmonary (SN-PTB), and extra-pulmonary (EP-TB)^24,27^. Pulmonary TB disease forms were assumed infectious, but at varying levels^24,28^.

Treated individuals without DM proceed to the recovery state in six months reflecting the typical 6-months treatment course under the directly-observed treatment, short course (DOTS)^29^. Treated and recovered individuals can be re-exposed to a new TB infection, and subsequently proceed to TB disease.

DM individuals followed a similar TB natural history to that of non-DM individuals, but the natural history was modulated by specific effects of having DM. DM was assumed to affect TB’s natural history and treatment outcomes at the different TB stages. Further details on the model can be found in Supplementary Text S1.

Model parameters were chosen according to empirical evidence and through model fitting to data. Supplementary Table S1 lists the parameter values and their sources. Data for India were used to parameterize the country-specific parameters^30,31^. India was chosen as an illustrative example since both TB incidence and DM prevalence are high in this country^32^. Note that given that we used arbitrary effect sizes for the interaction effects, this exploratory analysis does not provide estimates of the population impact of DM on TB in India.

The model was fitted to TB-incidence (i.e. 217 per 100,000 population per year), mortality (i.e. 39 per 100,000 population per year), and case fatality (i.e. 0.17) rates for the year 2015, as obtained from the WHO’s Global Health Observatory data repository^30^; and to national DM prevalence (i.e. 8.6%) for the year 2015, as obtained from the International Diabetes Federation^31^. Three country-specific parameters were derived by model fitting to data: respiratory contact rate, TB case detection rate, and DM-related mortality rate.

The population size was held constant to disentangle the TB-DM epidemiological effects from the demographic effects. All analyses were conducted assuming endemic equilibrium for TB. The model was coded and analyzed in MATLAB R2015a^33^.

### Epidemiologic implications of TB-DM interactions

Using the model, we assessed the implications of each of the plausible/potential DM effects on TB epidemiology, individually and in combinations. For a standardized comparison of the impact across the different mechanisms, we assumed a flat change of 300% for each pathway by which DM affects TB; that is, a standard effect size (ES) of 3.0 for each mechanism with an expected ES ≥1, and (an inverse) ES of 1/3 for each mechanism with an ES <1. This specific choice of ES value is relevant but otherwise arbitrary, as we aimed to assess the impact given a specific standardized ES for all DM-on-TB effects. This value of ES of 3.0 was deemed reasonable given the strength of the observed association (for different but closely-related statistical measures) between TB and DM in systematic reviews and meta-analyses^5,6,8^. Noting that, for most effects, the ES as estimated in the literature was heterogeneous in value, had only suggestive evidence with no assessed value, or could not be disentangled from the ES of another effect. While we used an ES of 3.0 for each effect, our model is general and can assess theoretically the impact of any combination of ESs.

We assessed the TB-DM epidemiologic synergy using two population-level measures: incidence hazard ratio (HR) and “true” population attributable fraction (*PAF*_*True*_). The HR was defined as the ratio of TB disease incidence rates among those with DM over those with no DM, within the same population (Equation 2.1 of Supplementary Text S1).

The *PAF*_*True*_ was assessed for three TB-disease outcome measures: incidence, prevalence, and mortality. For each, it was defined as the proportional reduction in the measure in a comparison between the measure in a scenario where there is TB-DM epidemiologic synergy (some active effects for DM on TB), compared to a counter-factual scenario where there is no TB-DM epidemiologic synergy (no active effect; Equation 2.2 of Supplementary Text S1). These two scenarios were simulated using the model by assuming an ES of 3.0 (or 1/3) in the epidemiologic-synergy scenario, and an ES of 1.0 in the no-epidemiologic-synergy scenario. This approach for assessing the *PAF* is labeled as “true” *PAF*^34,35^, because it captures the *direct* (etiological) effects of DM on TB, as well as the *indirect* (such as onward transmission) effects of DM on TB.

In addition to *PAF*_*True*_, we estimated the *PAF* using the conventional but simplistic *Levin’s formula* (*PAF*_*Levin*_)^23^, which cannot capture the indirect effects, for comparison purposes (Equation 2.3 of Supplementary Text S1). Strictly speaking, we assessed the *PAF*_*Levin*_ for only the specific situation that it applies: assuming that DM increases the risk of developing TB disease by a factor of 3 (relative risk (*RR*) = 3), based on existing estimates^5,6,8^ and not on a specific DM-on-TB effect.

## Results

### Conceptual framework for TB-DM interactions

Figure 2 illustrates the conceptual framework of TB-DM interactions that was developed based on the evidence gleaned from the literature review, for epidemiologically-relevant effects with either robust or suggestive evidence. DM was accordingly postulated to affect TB’s natural history and treatment outcomes in 10 different potential ways. Each of these was labeled as “effect” and numbered accordingly. A description of each effect and citations to its supporting evidence can be found in Table 1. Of notice that the strength of the evidence based varied for each effect, and quite often was either potentially biased, or estimated with wide confidence intervals.

**Table 1:** The plausible effects of diabetes mellitus (DM) on tuberculosis (TB) natural history and treatment outcomes.

Effect	Description	Statistical measure of effect as incorporated in the model	Expected range based on available evidence	Sources
**Effects of DM on TB’s natural history (TB infection and/or TB disease)**				
*Effect 1-Susceptibility*	DM increases the susceptibility to TB infection	Hazard ratio	≥1	^36– 40^
*Effect 2-Fast progression*	DM increases the proportion of TB infections entering latent-fast state as opposed to latent-slow state	Proportion ratio	≥1	5,8,10,42–48
*Effect 3-Reactivation*	DM increases the rate of developing TB disease among those with latent TB infection	Rate ratio	≥1	^5, 8, 10, 42– 48^
*Effect 4-Latent reinfection*	DM increases the susceptibility to TB reinfection among those with latent-slow TB infection	Hazard ratio	≥1	^5, 8, 10, 42– 48^
*Effect 5-Smear positivity*	DM increases the proportion of new PTB^#^ disease cases progressing to SP-PTB* as opposed to SN-PTB^$^	Proportion ratio	≥1	^51– 60^
*Effect 6-Disease infectiousness*	DM increases the infectiousness of PTB (SP-PTB and SN-PTB) for both untreated and treated TB disease cases	Factor ratio	≥1	^51, 54, 61, 62^
*Effect 7-TB mortality*	DM increases the hazard of TB-related mortality for both untreated and treated TB disease cases	Hazard ratio	≥1	^12, 52, 53, 60, 61, 63^
**Effects of DM on TB treatment outcomes**				
*Effect 8-Treatment failure*	DM reduces the proportion of successful treatment (through increased risk of treatment failure and MDR-TB^¥^)	Proportion ratio	≥1	^12, 57, 64^
*Effect 9-Recovery*	DM reduces the rate of TB recovery (i.e. prolongs the recovery time) for those who recover naturally or due to treatment	Rate ratio	≥1	^51, 54, 55, 64– 67^
*Effect 10-Cured reinfection*	DM increases the susceptibility to TB reinfection among those treated or recovered from TB disease	Hazard ratio	≥1	^12, 64^

^#^PTB: Pulmonary TB; *SP-PTB: smear-positive pulmonary TB; ^$^SN-PTB: smear-negative pulmonary TB; ^¥^MDR-TB: multi-drug resistant TB.

There was evidence suggesting that DM may increase *susceptibility to TB infection* (*Effect 1-Susceptibility*)^36–41^—an effect to be distinguished from that of increasing *susceptibility to TB disease*.

Ample evidence supported an increased risk of developing TB disease for those with DM^5,8,10,42–50^. However, this evidence did not differentiate the precise biological mechanism of whether DM is associated with increased proportion of TB infections entering the LFI state, as opposed to the LSI state (*Effect 2-Fast progression)*; increased susceptibility to develop TB disease among those with LSI (*Effect 3-Reactivation*); and/or increased susceptibility to TB reinfection among those with LSI (*Effect 4-Primary reinfection*).

Several studies indicated that DM increases the proportion of developing SP-PTB disease (as opposed to SN-PTB) among those who progress to pulmonary TB disease (*Effect 5-Smear positivity*)^51–60^. DM was also found to be a risk factor for increased *M*. *tuberculosis* bacterial load^51,54,61,62^; a proxy biomarker for increased TB infectiousness among those with pulmonary TB disease (*Effect 6-Disease infectiousness*)—that is, DM increasing the risk of TB transmission per one respiratory contact as a consequence of the higher bacterial load.

Strong evidence indicated that DM increases the risk of TB-related mortality among treated TB-disease individuals (*Effect 7-TB mortality*)^12,13,52,53,60,61,63^. We assumed, given biological plausibility, that the same effect applies for untreated TB disease individuals (*Effect 7-TB mortality*).

Several studies indicated that DM reduces the proportion of successful treatment among those undergoing TB treatment (through increased risk of treatment failure and multi-drug resistant TB; *Effect 8-Treatment failure*)^12,13,57,64^. Also, recent studies demonstrated a greater risk of persisting TB smear or sputum culture positivity by the second to third month of treatment, delaying the resolution of TB disease among those with DM compared to those without DM (*Effect 9-Recovery*)^51,54,55,64–67^.

Evidence indicated an increased susceptibility to TB reinfection after TB treatment and recovery among those with DM (*Effect 10-Cured reinfection*)^12,13,64^. Cured reinfection is defined as a subsequent episode of TB disease in a TB patient who received at least six months of TB treatment, but developed active TB after successful treatment (i.e. smear or sputum culture was negative at the end of the treatment period). Given biological plausibility, we assumed that the same effect applies to naturally recovered individuals.

All these effects were incorporated in the conceptual framework, and in the TB-DM mathematical model, as delineated in Equation 2.2 of Supplementary Text S1.

### Impact of TB-DM interactions on the hazard ratio of TB disease

**Figure 3:**
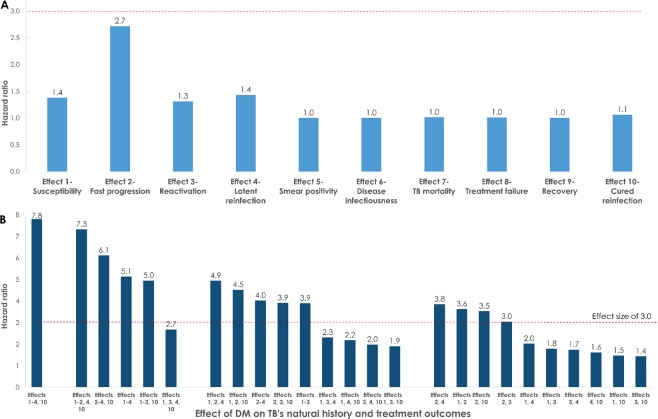
Epidemiological impact of the 10 plausible effects of diabetes mellitus (DM) on tuberculosis (TB) natural history and treatment outcomes, as measured by the incidence hazard ratio (HR) of TB disease among those with DM compared to those without DM. (**A**) Results of the impact of each of the effects individually. (**B**) Results of the impact of all possible combinations of the effects that individually had an HR >1.0. Each DM on TB effect had a standardized effect size (ES) of 3.0 if the expected ES (based on evidence) is ≥1, and (an inverse) ES of 1/3 if the expected ES is <1 (red line).

Figure 3A shows the model-estimated HRs for the 10 TB-DM effects. The standardized ES of 3.0 (or 1/3) for each effect impacted the HR differently. *Effect 2-Fast progression* had the largest impact with an HR of 2.7, which is within the range found in observational studies^8^. *Effect 3-Reactivation*, *Effect 1*-*Susceptibility*, and *Effect 4*-*Latent reinfection* had an intermediate HR impact in the range of 1.3–1.4, which is also within the range found in observational studies^8^. *Effect 10-Cured reinfection* had a minor HR impact of 1.1, which is outside the range found in observational studies^8^. For the remaining effects (*Effect* 5-*Smear positivity*, *Effect 6-Disease infectiousness*, *Effect 7-TB mortality*, *Effect 8-Treatment failure*, and *Effect 9-Recovery*), the HR was 1.0—these effects had no impact on the HR, which is also outside the range found in observational studies^8^.

By assessing all possible effect combinations (for those with HR >1.0), the HR ranged between 1.4 (*Effect 3-Reactivation* and *Effect 10-Cured reinfection*) and 7.8 (combining all effects; Fig. 3B). Every combination that included *Effect 2-Fast progression* reached an HR of 3.0 or higher, but none of the combinations that did not include *Effect 2-Fast progression* reached an HR of 3.0 or higher.

By assessing all possible pairwise combinations for all effects, the HR ranged between 1.0 (for several pairwise combinations) and 3.8 (*Effect 2-Fast progression* and *Effect 4*-*Latent reinfection*; Supplementary Fig. S1A). Few of the pairwise combinations that included *Effect 2-Fast progression* reached an HR of 3.0 or higher, but none of the combinations that did not include *Effect 2-Fast progression* reached an HR of 3.0 or higher.

### Variation in the effect size of each of the DM-on-TB effects and observed HR

**Figure 4:**
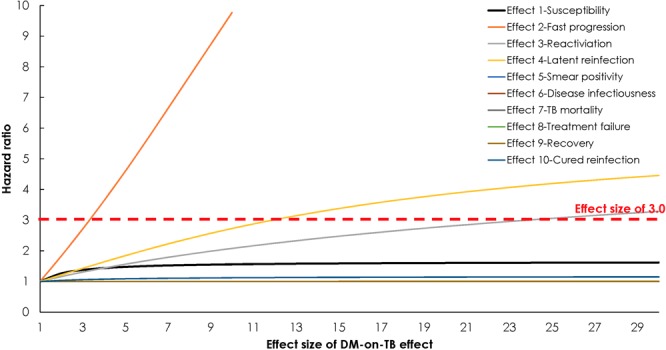
Assessment of varying the effect size of each of the diabetes mellitus (DM) on tuberculosis (TB) effects to yield the observed hazard ratio (HR) of 3.0. HR is defined as the ratio of TB disease incidence rate among those with DM compared to those without DM.

In an additional one-way sensitivity analysis, the ES of each of the DM-on-TB effects was varied to yield the observed HR of 3.0 (Fig. 4). For *Effect 1*-*Susceptibility*, *Effect* 5-*Smear positivity*, *Effect 6-Disease infectiousness*, *Effect 7-TB mortality*, *Effect 8-Treatment failure*, *Effect 9-Recovery*, and *Effect 10-Cured reinfection*, no value for the ES would yield an HR value of 3.0. However, for *Effect 2-Fast progression*, *Effect 3-Reactivation*, and *Effect 4*-*Latent reinfection* a value of 3.4, 24.0, and 12.0, respectively, would yield an HR value of 3.0. Moreover, the ranking of the impact on HR of the DM-on-TB effects barely changed regardless of the ES, appart from a minor change in the ranking of *Effect 1-Susceptibility*, mainly due to the saturation of the impact of this effect on the HR (Fig. 4). The sensitivity of the model output to changes in TB fast progression has been also observed in a previous modelling study^68^.

The epidemiological impact of varying simultaneously the ES (from 1.5 to 5.0) of all pairwise combinations of the effects that individually had an HR >1.0 (for an ES of 3.0) was assessed in an additional sensitivity analysis (Fig. S3). For ES less than 4.0, the ranking of the impact on HR for the DM-on-TB effects was unchanged. However, for higher ES, there was one main change in the ranking; the scale of the HR for the combination of *Effect 2-Fast progression* and *Effect 10-Cured reinfection* became higher than other combinations, highlighting the importance of their synergy, possibly related to surpassing the reinfection threshold^69–72^.

### Impact of TB-DM interactions on the population attributable fractions

**Figure 5:**
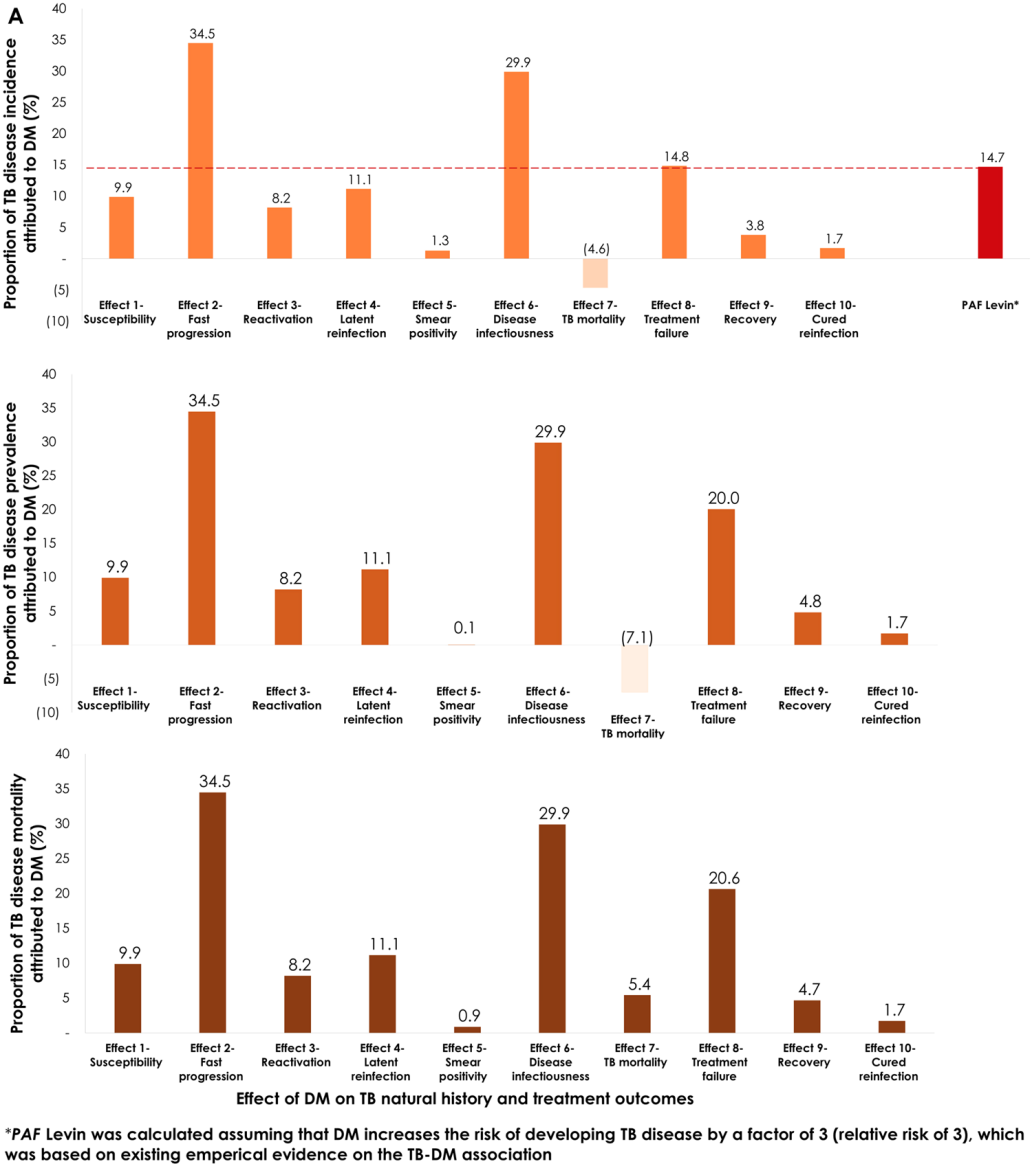
Proportion of tuberculosis (TB) disease incidence (**A**), prevalence (**B**), and mortality (**C**) attributed to each of the effects of diabetes mellitus (DM) on TB natural history and treatment outcomes. These population attributable fraction (*PAF*_*True*_) measures were estimated as the proportional reduction in the measure in a comparison between the measure in a scenario where there is TB-DM epidemiologic synergy (that is some effect for DM on TB is active), compared to a counter-factual scenario where there is no TB-DM epidemiologic synergy. Each DM on TB effect had a standardized effect size (ES) of 3.0 if the expected ES (based on evidence) is ≥1, and (an inverse) ES of 1/3 if the expected ES is <1. The red bar (and line) in panel **A** is the estimated *Levin’s formula* population attributable fraction, assuming a relative risk (*RR*) of 3.0 for TB-disease incidence among DM versus non-DM individuals.

Figure 5A shows the proportion of TB-disease incidence attributed directly or indirectly (i.e. *PAF*_*True*_) to DM for the 10 TB-DM effects assuming a standardized ES of 3.0 (or 1/3). *PAF*_*True*_ for TB-disease incidence ranged between −4.6% (*Effect 7-TB mortality*) and 34.5% (*Effect 2-Fast progression*). The second largest *PAF*_*True*_ at 29.9% was for *Effect 6-Disease infectiousness* (DM increasing TB infectiousness by three-fold). The lowest *positive PAF*_*True*_ at 1.3% was for *Effect* 5-*Smear positivity*.

Of notice, the impact of *Effect 5-Smear positivity* and *Effect 6-Disease infectiousness* on TB transmission dynamics were different, despite the apparent similarity in mechanism of action. *Effect 6-Disease infectiousness* increased TB transmission in the population by increasing the risk of TB transmission for a given infectious contact. While *Effect 5-Smear positivity* also led to increased TB transmission in the population by increasing the pool of smear-positive TB cases, this effect was undermined by the fact that smear-positive TB cases had a higher risk of TB mortality (Supplementary Table S1).

The effect of DM on TB mortality (*Effect 7-TB mortality*) caused a negative *PAF*_*True*_ of −4.6%, as the higher mortality of individuals with TB-DM reduced TB transmission in the population, and thus TB disease incidence (i.e. TB-DM individuals died before spreading the infection further).

The predicted impact of DM on TB-disease incidence using *Levin’s formula* (assuming that DM increases the risk of developing TB disease by three-fold) was estimated at 14.7% (Fig. 5A). The latter should be seen as a baseline for comparison of the different impacts.

Figure 5B shows the proportion of TB-disease prevalence attributed to DM for the 10 effects, assuming similary ES of 3.0 (or 1/3). The impact of each effect on TB-disease prevalence was overall similar to that on TB-disease incidence (Fig. 5B). The proportion of TB-disease prevalence attributed to each effect ranged between −7.1% (*Effect 7-TB mortality*) and 34.5% (*Effect 2-Fast progression*).

Figure 5C shows the proportion of TB-disease mortality attributed to DM for the 10 effects, assuming similary ES of 3.0 (or 1/3). The pattern was overall similar to that for TB-disease incidence and prevalence. The proportion of TB-disease mortality attributed to each effect ranged between 0.9% (*Effect* 5-*Smear positivity*) and 34.5% (*Effect 2-Fast progression*). The effect on TB mortality (*Effect 7-TB mortality*) caused here a *positive* impact of 5.4%, versus the *negative* impacts on TB-disease incidence (−4.6%) and prevalence (−7.1%).

Supplementary Figure S1 shows the results for *PAF*_*True*_ for all possible combinations of the five effects that individually had the largest *PAF*_*True*_. The proportion of TB-disease incidence attributed to DM ranged between 22.5% (*Effect 1-Susceptibility* and *Effect 4-Latent reinfection*) and 89.1% (combining all effects). By assessing all possible pairwise combinations for all effects, the *PAF*_*True*_ for incidence ranged between −3.5% (*Effect 7-TB mortality* and *Effect 10-Cured reinfection*) and 70.9% (*Effect 2-Fast progression* and *Effect 6-Disease infectiousness*; Supplementary Fig. S2B).

Table 2 shows a comparison of the impact of DM on TB-disease incidence as measured by *PAF*_*True*_ and HR. *Effect 2-Fast progression* had the highest impact using both measures. There were some effects that had large impact on *PAF*_*True*_, but limited or no impact on HR, most notably *Effect 6-Disease infectiousness* that had the second largest *PAF*_*True*_.

**Table 2:** The epidemiologic implications* of each of the plausible diabetes mellitus (DM) effects on tuberculosis (TB) natural history and treatment outcomes as measured by the “true” population attributable fraction (*PAF*_*True*_) and incidence hazard ratio (HR).

Effect^#^	*PAF*_*True*_	HR
*Effect 2-Fast progression*	34.5%	2.7
*Effect 6-Disease infectiousness*	29.9%	1.0
*Effect 8-Treatment failure*	14.8%	1.0
*Effect 4-Latent reinfection*	11.1%	1.4
*Effect 1-Susceptibility*	9.9%	1.4
*Effect 3-Reactivation*	8.2%	1.3
*Effect 9-Recovery*	3.8%	1.0
*Effect 10-Cured reinfection*	1.7%	1.1
*Effect 5-Smear positivity*	1.3%	1.0
*Effect 7-TB mortality*	−4.6%	1.0
If no effect of DM on TB	0.0%	1.0
Relevant reference measure	14.7%^$^	3.0^€^

^#^Effects are ordered from largest to lowest *PAF*. ^$^*PAF* estimated using *Levin’s formula*^23^. ^€^Typical effect size using different, but closely-related statistical measures (such as hazard ratio, relative risk, rate ratio, and odds ratio) of the strength of the observed TB-DM association^5,6,8^.

*We assumed a standard effect size of 3.0 for each mechanism with an expected effect size ≥1 and (an inverse) effect size of 1/3 for each mechanism with an effect size ≤1.

## Discussion

We investigated the mechanisms by which DM can affect TB natural history and treatment outcomes, and therefore can impact TB-transmission dynamics. Seven epidemiologically-relevant plausible effects for DM on TB natural history, and three for DM on TB treatment outcomes, were identified based on literature review. Informed by this empirical evidence, we developed a conceptual framework of DM’s effects on TB (Fig. 2), and translated it into a mathematical model to investigate the impact of these effects on TB-transmission dynamics. Our main findings show that conventional estimates of the *PAF* (that is using *Levin’s formula*) indicate that 15% of TB-disease incidence is attributable to DM (in India where the model was applied for *Effect 2-Fast progression*; Fig. 5A), but taking into account indirect effects (onward transmission), the “true” *PAF* could be two-fold higher at 35%. The “true” *PAF*, however, depends on which DM-on-TB effect (or combination of effects) is assumed to be active; looking at each listed pathway individually and assuming a standardized ES of 3.0 (or 1/3) yielded a “true” *PAF* that ranged from −5% to 35% (Fig. 5).

Although the 10 effects impacted TB-transmission dynamics (Fig. 5), the impact (for several of them) could not be captured by the HR (Fig. 3A)—the conventional epidemiologic measure of the TB-DM association. The reason is that the HR captures only the effects of DM on *directly developing TB disease*, but cannot capture effects that leads *indirectly* to more TB disease in the population. For example, evidence suggests a higher TB infectiousness with DM (*Effect 6-Disease infectiousness*), because of higher *M*. *tuberculosis* bacterial load^51,54,61,62^. The higher infectiousness contributes to more TB transmission in the population, but this effect cannot be captured by a study that compares TB incidence among those with DM to those without DM (conventional cohort or case-control studies)—the higher infectiousness affects *both comparison groups simultaneously*.

Of the 10 investigated effects, only four resulted in an impact that could be actually measured by the HR (Fig. 3A). Even though the ES of each effect was standardized at 3.0, all effects had an HR <3; the HR was not a representative measure of the *true* ES of each effect. *Effect 2-Fast progression* resulted in the largest HR at 2.7, while *Effect 3-Reactivation*, *Effect 1*-*Susceptibility*, and *Effect 4*-*Latent reinfection* had HRs of only about 1.4. Also, based on the comparison between the measured HR assuming the standard ES of 3.0 and the actual pooled evidence for the TB-DM assosciation, including prospective (3.59, 95% confidence interval [CI] 2.25–5.73), retrospective (1.55, 95% CI 1.39–1.72), and case-control studies (2.09, 95% CI 1.71–2.55); it seems that only *Effect 1*-*Susceptibility*, *Effect 2-Fast progression*, *Effect 3-Reactivation*, and *Effect 4*-*Latent reinfection* can explain the TB-DM assosiation.

It is possible that the 10 TB-DM effects could be acting simultanously, and therefore their individual impacts on the HR are difficult (if not impossible) to disentangle. Figure 4 highlights that some pathways are impossible to result in an HR of 3.0 by themselves (i.e. *Effect 1*-*Susceptibility*, *Effect* 5-*Smear positivity*, *Effect 6-Disease infectiousness*, *Effect 7-TB mortality*, *Effect 8-Treatment failure*, *Effect 9-Recovery*, and *Effect 10-Cured reinfection*), while other pathways can reach an HR of 3.0 by themselves but only at very high effect sizes (i.e. effect size of 24 and 12 for *Effect 3-Reactivation* and *Effect 4*-*Latent reinfection*, respectively).

The measured HR in an epidemiological study may reflect thus the *combined* effect of several individual effects (Fig. 3B and Supplementary Fig. S2A). For instance, by combining *Effect 2-Fast progression* and *Effect 3-Reactivation*, the HR of 3.0 (suggested by the recently updated systematic review^7^) can be easily reached (Fig. 3B and Supplementary Fig. 4). Indeed, some combinations that included *Effect 2-Fast progression* reached an HR of 3.0 or higher, but none of the combinations that did not include *Effect 2-Fast progression* reached an HR of 3.0 or higher. Even for some of the effects that alone did not have an HR >1 (e.g. *Effect 6-Disease infectiousness*, *Effect 7-TB mortality*, and *Effect 8-Treatment failure*), by combining them with *Effect 2-Fast progression* an HR higher than the HR of *Effect 2-Fast progression* alone was reached, higlighting synergy in combining these effects (Supplementary Fig. S2A). Supplementary Figure S3 also highlights the potential synergy in combining effects, and shows that the impact (i.e. ranking/importance) of some of the pairwise combinations of the TB-on-DM effects on HR may change at large values for the ES (here larger than 4.0).

These findings highlight how the focus on measures such as HR, relative risk, rate ratio, and odds ratio, to assess the TB-DM association, can be misleading. These measures cannot capture the actual impact of some TB-DM effects which influence TB epidemiology indirectly—the focus on such measures may be preventing us from appreciating the extent to which DM is influencing TB-transmission dynamics.

We found that the impact of DM on TB is better assessed using the “true” *PAF* (*PAF*_*True*_) as it captures both the direct and indirect impacts, and for all 10 effects. Taking *Effect 6-Disease infectiousness* (that had an HR = 1.0; Fig. 3A) as an example, 30% of TB-disease incidence was attributed to this effect (assuming three-fold increased TB infectiousness with DM; Fig. 5A). This large impact arises not only from the *direct* enhanced TB transmission from those with concurrent DM, but also from the *indirect* onward transmission with the larger pool of infected persons in the population.

Of all effects, assuming a standard ES, *Effect 2-Fast progression* had the largest impact (Fig. 5). This effect increases TB-disease incidence by increasing the fraction of infections that progress rapidly to TB disease (*direct impact*), but also increases (*indirect impact*) the onward transmission and circulation of TB among both DM and non-DM persons. It is striking that the indirect impact was comparable in scale to the direct impact. This can be seen by comparing the *PAF*_*True*_ of 35% to that of *PAF*_*Levin*_ of only 15% (the latter measures only the direct impact on incidence). These findings further demonstrate how the conventional approach to assess the *PAF* due to DM using *PAF*_*Levin*_^14,17–21^, could be underestimating the extent to which DM is influencing TB-transmission dynamics. This, however, will depend on which of the DM-on-TB effects (or combination of effects) is assumed active, as each effect may impact TB transmission dynamics differently, with *PAF*_*True*_ ranging from −5% to 35% (Fig. 5).

Though only two effects had large *PAF*_*True*_, most effects had small *PAF*_*True*_ (Fig. 5). The combined effect, however, even of small effects, can add up to a substantial impact (Supplementary Fig. S1). If several of the 10 effects (large and small) are present and acting simultanously, the impact of DM on TB could be substantially higher than expected (Fig. 5 and Supplementary Fig. S2B). This highlights the need to ascertain the exact ES of each effect, and suggests that DM could be impacting TB epidemiology to a larger extent than previously thought.

Our study has limitations. We included the plausible TB-DM effects based on a literature review, but we may have overlooked effects, particularly if they are not yet supported by evidence. Though there is evidence supporting each considered effect, the evidence is not conclusive for most, and the ES is either imprecise or poorly known. We did not include all factors that may influence the incorporated DM-on-TB effects, or the factors that may affect directly each of TB or DM burdens individually^8,63,73,74^. For example, we did not incorporate the impact of using anti-DM medications^75–77^, or HIV as a co-factor^63,73,74^. However, in India (as in other parts of the world) a large proportion of people living with DM are undiagnosed and may have uncontrolled DM^8,78–80^. Moreover, even for those diagnosed with DM, a proportion of them may not be adhering to anti-DM medications^81^. Despite the potential public health implications, prevalence of HIV is relatively low in India at less than 1.0%^82^, a fact that is true for nearly all TB-DM burdened countries outside Africa^83^, hence, minimally affecting our results and conclusions.

We modeled TB’s natural history based on the canonical approach in the literature^24,84^, but TB has a complex natural history that is still far from being settled^68,85^. We assessed the TB-DM epidemiologic synergy assuming endemic and stable levels for TB and DM, with no assessment of the implications of the temporal-dynamics and time-varying infectivity profile. The DM-on-TB effects were assumed constant with time, but the fluctuating blood glucose levels in individuals with DM may affect the stability of the rates as assumed in this model. The model did not include the varying age-stratification for DM, but DM is strongly age-dependent. Bearing these limitations in mind, the aim of the present analysis was to assess the epidemiological implications of the TB-DM interactions from a theoretical perspective that focuses on the core interaction effects, and avoids entanglement with demographic and temporal effects. Thus, we resorted to a parsimonious model structure where DM is included as a fixed proportion of the population and with temporally-invariable DM-on-TB effects, thereby presenting an “average” impact of DM on TB rather than a full temporally-varying impact.

We did not explicitly factor multi-drug resistant TB (MDR TB) and the effect of DM on MDR TB in the model. However, <5% of newly treated TB cases globally are estimated to have MDR TB^86^, and thus this is not likely to affect our results. We did not factor the effect of intermediate hyperglycemia (pre-DM) on TB, leading to plausible underestimation of DM’s impact on TB. We focused on the effects of DM on TB, but the links between the two diseases could be bi-directional^46^, thereby further complicating analyses of their epidemiologic synergy.

In conclusion, we provided a conceptual mapping of how DM affects TB natural history and treatment outcomes through 10 plausible effects, and investigated the epidemiological impact of each effect on TB-transmission dynamics. We used a standardized ES for each effect though in reality each may vary with implications on the HR and the *PAF*. The ESs of these effects are yet to be established with precision, and therefore we cannot determine nor draw specific conclusions about the exact and total impact of DM on TB in India. Several effects could not be assessed using conventional epidemiologic-study designs of the TB-DM association, and therefore their impact may have been overlooked in existing literature. The impact of DM on TB should be assessed using a *PAF*_*True*_ approach, as the one presented here, since this approach can capture the combined direct and indirect impacts of each effect. Thus, the unique contribution of our paper is to highlight the potentially large indirect (true population) effects associated with some pathways, particularly if DM’s effects on TB are dominated by rapid TB progression or increased infectiousness.

We found that the indirect impact on TB-transmission dynamics (e.g. onward transmission) of some of the effects is large and comparable to the direct impact. Even for effects with small impacts, the combined effect of several could be substantial. While the impact of several effects on the HR was limited, the impact on the *PAF* was substantial suggesting that DM could be impacting TB epidemiology to a larger extent than previously thought. They also stress the need to assess with precision the ESs of these effects to determine the actual total impact of DM on TB. A better understanding of the TB-DM epidemiologic synergy is critical to improved control and preventive strategies for TB disease burden, and to achieving the goal of TB elimination by 2050.

## Supplementary information

Supplementary Information

## References

[1] World Health Organization. Global tuberculosis report 2018 (Available from, http://apps.who.int/iris/bitstream/handle/10665/274453/9789241565646-eng.pdf?ua=1, accessed Sept. 2018) (2018).

[2] World Health Organization. Global tuberculosis report 2016 (Available from, http://apps.who.int/iris/bitstream/10665/250441/1/9789241565394-eng.pdf?ua=1, accessed on May 2017) (2016).

[3] World Health Organization. Draft global strategy and targets for tuberculosis prevention, care and control after 2015 (Available at, http://apps.who.int/gb/ebwha/pdf_files/WHA67/A67_11-en.pdf?ua=1) (March 2014).

[4] Boucot KR (1957). Diabetes mellitus and pulmonary tuberculosis. J Chronic Dis.

[5] Jeon CY, Murray MB (2008). Diabetes mellitus increases the risk of active tuberculosis: a systematic review of 13 observational studies. PLoS medicine.

[6] World Health Organization & International Union Against Tuberculosis and Lung Disease. Collaborative framework for care and control of tuberculosis and diabetes. (World Health Organization, Switzerland, 2011).

[7] International Diabetes Federation. IDF Diabetes Atlas. Eighth edition. Brussels, Belgium (Available at, http://www.diabetesatlas.org, accessed Dec. 2017) (2017).

[8] Al-Rifai RH, Pearson F, Critchley JA, Abu-Raddad LJ (2017). Association between diabetes mellitus and active tuberculosis: A systematic review and meta-analysis. PLoS One.

[9] Faurholt-Jepsen D (2013). Diabetes is a strong predictor of mortality during tuberculosis treatment: a prospective cohort study among tuberculosis patients from Mwanza, Tanzania. Tropical medicine & international health: TM & IH.

[10] Stevenson CR (2007). Diabetes and the risk of tuberculosis: a neglected threat to public health?. Chronic illness.

[11] Faurholt-Jepsen D (2012). The role of diabetes co-morbidity for tuberculosis treatment outcomes: a prospective cohort study from Mwanza, Tanzania. BMC infectious diseases.

[12] Baker MA (2011). The impact of diabetes on tuberculosis treatment outcomes: a systematic review. BMC medicine.

[13] Huangfu, P., Ugarte-Gil, C., Golub, J., Pearson, F. & Critchley, J. The effects of diabetes on tuberculosis treatment outcomes: an updated systematic review and meta-analysis. *Under Review* (2017). 10.5588/ijtld.18.0433 31439109

[14] Ruslami R, Aarnoutse RE, Alisjahbana B, van der Ven AJ, van Crevel R (2010). Implications of the global increase of diabetes for tuberculosis control and patient care. Tropical medicine & international health: TM & IH.

[15] Lonnroth K, Jaramillo E, Williams BG, Dye C, Raviglione M (2009). Drivers of tuberculosis epidemics: the role of risk factors and social determinants. Social science & medicine (1982).

[16] Harries AD (2013). Epidemiology and interaction of diabetes mellitus and tuberculosis and challenges for care: a review [Review article]. Public Health Action.

[17] Stevenson CR (2007). Diabetes and tuberculosis: the impact of the diabetes epidemic on tuberculosis incidence. BMC public health.

[18] Lonnroth K (2010). Tuberculosis control and elimination 2010-50: cure, care, and social development. Lancet.

[19] Odone A, Houben RM, White RG, Lonnroth K (2014). The effect of diabetes and undernutrition trends on reaching 2035 global tuberculosis targets. The lancet. Diabetes & endocrinology.

[20] Walker C, Unwin N (2010). Estimates of the impact of diabetes on the incidence of pulmonary tuberculosis in different ethnic groups in England. Thorax.

[21] Pan SC (2015). Effect of diabetes on tuberculosis control in 13 countries with high tuberculosis: a modelling study. *The lancet*. Diabetes & endocrinology.

[22] Harries AD (2010). Defining the research agenda to reduce the joint burden of disease from diabetes mellitus and tuberculosis. Tropical medicine & international health: TM & IH.

[23] Levin ML (1953). The occurrence of lung cancer in man. Acta Unio Int Contra Cancrum.

[24] Abu-Raddad LJ (2009). Epidemiological benefits of more-effective tuberculosis vaccines, drugs, and diagnostics. Proceedings of the National Academy of Sciences of the United States of America.

[25] Vynnycky E, Fine PE (2000). Lifetime risks, incubation period, and serial interval of tuberculosis. Am J Epidemiol.

[26] Sutherland I, Svandova E, Radhakrishna S (1982). The development of clinical tuberculosis following infection with tubercle bacilli. 1. A theoretical model for the development of clinical tuberculosis following infection, linking from data on the risk of tuberculous infection and the incidence of clinical tuberculosis in the Netherlands. Tubercle.

[27] Dye C, Garnett GP, Sleeman K, Williams BG (1998). Prospects for worldwide tuberculosis control under the WHO DOTS strategy. Directly observed short-course therapy. Lancet.

[28] Small PM (1994). The epidemiology of tuberculosis in San Francisco. A population-based study using conventional and molecular methods. The New England journal of medicine.

[29] World Health Organization. The Global Plan to Stop TB 2011-2015 (available at, http://www.stoptb.org/assets/documents/global/plan/tb_globalplantostoptb2011-2015.pdf) (2011–2015).

[30] World Health Organization. WHO Global Health Observatory Data Repository, (available at, http://apps.who.int/gho/data/node.main), (2017).

[31] International Diabetes Federation. *IDF Diabetes Atlas*. 7th edition. Brussels, Belgium (Available at, http://www.diabetesatlas.org; accessed on September 2016) (2016).

[32] International Diabetes Federation. I*DF diabetes atlas*, sixth edition (available at, www.idf.org/diabetesatlas). (International Diabetes Federation, 2013).

[33] The language of technical computing v. 8.5.0.197613 (R2015a). Natick, MA, USA: (The MathWorks, Inc., 2018).

[34] Orroth KK (2006). Empirical observations underestimate the proportion of human immunodeficiency virus infections attributable to sexually transmitted diseases in the Mwanza and Rakai sexually transmitted disease treatment trials: Simulation results. Sex Transm Dis.

[35] Abu-Raddad LJ (2008). Genital herpes has played a more important role than any other sexually transmitted infection in driving HIV prevalence in Africa. PLoS One.

[36] Jackson C (2013). S57 Diabetes and latent tuberculosis infection: nested case-control study within the PREDICT cohort. Thorax.

[37] Martinez-Aguilar G (2015). Associated Risk Factors for Latent Tuberculosis Infection in Subjects with Diabetes. Arch Med Res.

[38] Brock I (2006). Latent tuberculosis in HIV positive, diagnosed by the M. tuberculosis specific interferon-gamma test. Respiratory research.

[39] Webb EA (2009). High prevalence of Mycobacterium tuberculosis infection and disease in children and adolescents with type 1 diabetes mellitus. The international journal of tuberculosis and lung disease: the official journal of the International Union against Tuberculosis and Lung Disease.

[40] Chan-Yeung M (2006). Prevalence and determinants of positive tuberculin reactions of residents in old age homes in Hong Kong. The international journal of tuberculosis and lung disease: the official journal of the International Union against Tuberculosis and Lung Disease.

[41] Lee MR (2017). Diabetes Mellitus and Latent Tuberculosis Infection: A Systemic Review and Metaanalysis. Clinical infectious diseases: an official publication of the Infectious Diseases Society of America.

[42] Baker MA, Lin H-H, Chang H-Y, Murray MB (2012). The Risk of Tuberculosis Disease Among Persons With Diabetes Mellitus: A Prospective Cohort Study. Clinical Infectious Diseases.

[43] Kim SJ, Hong YP, Lew WJ, Yang SC, Lee EG (1995). Incidence of pulmonary tuberculosis among diabetics. Tubercle and Lung Disease.

[44] Dobler Claudia Caroline, Flack Jeffrey Ronald, Marks Guy Barrington (2012). Risk of tuberculosis among people with diabetes mellitus: an Australian nationwide cohort study. BMJ Open.

[45] Leung CC (2008). Diabetic Control and Risk of Tuberculosis: A Cohort Study. American Journal of Epidemiology.

[46] Young F, Wotton CJ, Critchley JA, Unwin NC, Goldacre MJ (2012). Increased risk of tuberculosis disease in people with diabetes mellitus: record-linkage study in a UK population. Journal of epidemiology and community health.

[47] Shah BR, Hux JE (2003). Quantifying the risk of infectious diseases for people with diabetes. Diabetes care.

[48] Ponce-De-Leon A (2004). Tuberculosis and diabetes in southern Mexico. Diabetes care.

[49] Kamper-Jorgensen Z (2015). Diabetes-related tuberculosis in Denmark: effect of ethnicity, diabetes duration and year of diagnosis. The international journal of tuberculosis and lung disease: the official journal of the International Union against Tuberculosis and Lung Disease.

[50] Koesoemadinata RC (2017). Latent TB infection and pulmonary TB disease among patients with diabetes mellitus in Bandung, Indonesia. Transactions of The Royal Society of Tropical Medicine and Hygiene.

[51] Chiang CY (2015). The Influence of Diabetes, Glycemic Control, and Diabetes-Related Comorbidities on Pulmonary Tuberculosis. PLoS ONE.

[52] Wang JY, Lee LN, Hsueh PR (2005). Factors changing the manifestation of pulmonary tuberculosis. The international journal of tuberculosis and lung disease: the official journal of the International Union against Tuberculosis and Lung Disease.

[53] Wang CS (2009). Impact of type 2 diabetes on manifestations and treatment outcome of pulmonary tuberculosis. Epidemiology and infection.

[54] Singla R (2006). Influence of diabetes on manifestations and treatment outcome of pulmonary TB patients. The international journal of tuberculosis and lung disease: the official journal of the International Union against Tuberculosis and Lung Disease.

[55] Park SW (2012). The effect of diabetic control status on the clinical features of pulmonary tuberculosis. European journal of clinical microbiology & infectious diseases: official publication of the European Society of Clinical Microbiology.

[56] Chang JT (2011). Effect of type 2 diabetes mellitus on the clinical severity and treatment outcome in patients with pulmonary tuberculosis: a potential role in the emergence of multidrug-resistance. Journal of the Formosan Medical Association = Taiwan yi zhi.

[57] Alisjahbana B (2007). The effect of type 2 diabetes mellitus on the presentation and treatment response of pulmonary tuberculosis. Clinical infectious diseases: an official publication of the Infectious Diseases Society of America.

[58] Magee M (2015). Diabetes mellitus is associated with cavities, smear grade, and multidrug-resistant tuberculosis in Georgia. The International Journal of Tuberculosis and Lung Disease.

[59] Hongguang C (2015). Impact of diabetes on clinical presentation and treatment outcome of pulmonary tuberculosis in Beijing. Epidemiology & Infection.

[60] Magee MJ (2014). Diabetes mellitus and risk of all-cause mortality among patients with tuberculosis in the state of Georgia, 2009–2012. Annals of epidemiology.

[61] Dooley KE, Tang T, Golub JE, Dorman SE, Cronin W (2009). Impact of Diabetes Mellitus on Treatment Outcomes of Patients with Active Tuberculosis. The American Journal of Tropical Medicine and Hygiene.

[62] Restrepo BI (2007). Type 2 diabetes and tuberculosis in a dynamic bi-national border population. Epidemiology and infection.

[63] Faurholt-Jepsen D (2013). Diabetes is a strong predictor of mortality during tuberculosis treatment: a prospective cohort study among tuberculosis patients from Mwanza, Tanzania. Tropical Medicine & International Health.

[64] Jimenez-Corona ME (2013). Association of diabetes and tuberculosis: impact on treatment and post-treatment outcomes. Thorax.

[65] Perez-Navarro LM, Fuentes-Dominguez FJ, Zenteno-Cuevas R (2015). Type 2 diabetes mellitus and its influence in the development of multidrug resistance tuberculosis in patients from southeastern Mexico. Journal of diabetes and its complications.

[66] Restrepo BI (2008). Mycobacterial clearance from sputum is delayed during the first phase of treatment in patients with diabetes. The American journal of tropical medicine and hygiene.

[67] Viswanathan V (2014). Effect of diabetes on treatment outcome of smear-positive pulmonary tuberculosis–a report from South India. Journal of diabetes and its complications.

[68] Dowdy DW, Dye C, Cohen T (2013). Data needs for evidence-based decisions: a tuberculosis modeler’s ‘wish list’. The international journal of tuberculosis and lung disease: the official journal of the International Union against Tuberculosis and Lung Disease.

[69] Gomes MGM, Franco AO, Gomes MC, Medley GF (2004). The reinfection threshold promotes variability in tuberculosis epidemiology and vaccine efficacy. Proceedings. Biological sciences.

[70] Abu-Raddad LJ, van der Ventel BI, Ferguson NM (2008). Interactions of multiple strain pathogen diseases in the presence of coinfection, cross immunity, and arbitrary strain diversity. Physical review letters.

[71] Abu-Raddad LJ, Ferguson NM (2004). The impact of cross-immunity, mutation and stochastic extinction on pathogen diversity. Proc Biol Sci.

[72] Abu-Raddad LJ, Ferguson NM (2005). Characterizing the symmetric equilibrium of multi-strain host-pathogen systems in the presence of cross immunity. Journal of mathematical biology.

[73] Young F, Critchley JA, Johnstone LK, Unwin NC (2009). A review of co-morbidity between infectious and chronic disease in Sub Saharan Africa: TB and diabetes mellitus, HIV and metabolic syndrome, and the impact of globalization. Globalization and health.

[74] Levitt NS, Bradshaw D (2006). The impact of HIV/AIDS on Type 2 diabetes prevalence and diabetes healthcare needs in South Africa: projections for 2010. Diabet Med.

[75] Degner NR, Wang JY, Golub JE, Karakousis PC (2018). Metformin Use Reverses the Increased Mortality Associated With Diabetes Mellitus During Tuberculosis Treatment. Clinical infectious diseases: an official publication of the Infectious Diseases Society of America.

[76] Lee YJ (2018). The effect of metformin on culture conversion in tuberculosis patients with diabetes mellitus. Korean J Intern Med.

[77] Tseng Chin-Hsiao (2018). Metformin Decreases Risk of Tuberculosis Infection in Type 2 Diabetes Patients. Journal of Clinical Medicine.

[78] Critchley JA (2018). Glycemic Control and Risk of Infections Among People With Type 1 or Type 2 Diabetes in a Large Primary Care Cohort Study. Diabetes care.

[79] Shewade HD (2017). Effect of glycemic control and type of diabetes treatment on unsuccessful TB treatment outcomes among people with TB-Diabetes: A systematic review. PLoS One.

[80] Mahishale V (2017). Effect of Poor Glycemic Control in Newly Diagnosed Patients with Smear-Positive Pulmonary Tuberculosis and Type-2 Diabetes Mellitus. Iran J Med Sci.

[81] García-Pérez L-E, Alvarez M, Dilla T, Gil-Guillén V, Orozco-Beltrán D (2013). Adherence to therapies in patients with type 2 diabetes. Diabetes therapy: research, treatment and education of diabetes and related disorders.

[82] Paranjape RS, Challacombe SJ (2016). HIV/AIDS in India: an overview of the Indian epidemic. Oral diseases.

[83] UNAIDS. UNAIDS data 2018 (Available at, http://www.unaids.org/sites/default/files/media_asset/unaids-data-2018_en.pdf; Accessed Jan. 2019) (2018).

[84] Vynnycky E, Fine PE (1997). The natural history of tuberculosis: the implications of age-dependent risks of disease and the role of reinfection. Epidemiology and infection.

[85] Nico, J. D. Nagelkerke. *Courtesans and consumption*. *How sexually transmitted infections drive tuberculosis epidemics*. (Eburon, Delft. ISBN: 978-90-5972-603-1 (paperback), ISBN: 978-90-5972-604-8 (ebook), 2012).

[86] World Health Organization. Multidrug-resistant tuberculosis (MDR-TB) (available at, http://www.who.int/tb/challenges/mdr/MDR-RR_TB_factsheet_2017.pdf?ua=1, Accessed August, 2018) (World Health Organization, 2017).

