# Supplementary material for: Analytical Exploration of Potential Pathways by which Diabetes Mellitus Impacts Tuberculosis Epidemiology
Source: Sci Rep. 2019 Jun 11;9:8494. doi: 10.1038/s41598-019-44916-7 (PMC6560095; doi:10.1038/s41598-019-44916-7)
Supplement: Supplementary file 1 — Supplementary Information [file 41598_2019_44916_MOESM1_ESM.doc]

**Supplementary File**

**Analytical Exploration of Potential Pathways by which Diabetes Mellitus Impacts Tuberculosis Epidemiology**

Susanne F. Awad,^1,2^ Soha R. Dargham,^1^ Ryosuke Omori,^1,3,4,5^ Fiona Pearson,^2^ Julia A Critchley,^2^ and Laith J. Abu-Raddad,^1,5,6*^

^1^Infectious Disease Epidemiology Group, Weill Cornell Medicine-Qatar, Cornell University, Qatar Foundation - Education City, Doha, Qatar

^2^Population Health Research Institute, St George’s, University of London, London, UK

^3^Division of Bioinformatics, Research Center for Zoonosis Control, Hokkaido University, Sapporo, Hokkaido, Japan

^4^Japan Science and Technology Agency, PRESTO, Kawaguchi, Saitama, Japan

^5^Department of Healthcare Policy and Research, Weill Cornell Medicine, Cornell University, New York, New York, USA

^6^College of Health and Life Sciences, Hamad bin Khalifa University, Qatar Foundation, Education City, Doha, Qatar

**TEXT S1**

1. **Description of the Mathematical Modelling and Quantitative Approach**

**1.1 Overview**

We constructed a population-level deterministic mathematical model of tuberculosis (TB) transmission dynamics as an adaptation of an earlier published TB model^1^. We subsequently extended the model to include diabetes mellitus (DM) and the postulated effects of DM on TB.

The model incorporated two TB natural histories depending on the presence or absence of DM. TB natural history was described by the progression states of 1) susceptible, 2) latent TB infection, 3) TB disease, 4) treated TB disease, and 5) recovered.

*TB transmission dynamics in absence of DM*

For DM-free individuals (Equation 1.1), individuals are born susceptible ($S$), and remain susceptible but at risk of TB infection at a rate $\lambda$ (force of infection; Equation 1.3).

Upon TB infection, a proportion $p$ of the individuals pass to the stage of TB latent fast progression ($L^{F}$), and a proportion $1-p$ pass to the stage of latent slow progression ($L^{S}$). These two stages represent the epidemiologically observed fast and slow progression stages to TB disease^1^. Individuals in the TB latent-fast state remain as such but at risk of progression to TB disease at a (rapid) rate $\omega_{LF}$. Individuals in the TB latent-slow state remain as such but at risk of progression to TB disease at a (slow) rate $\omega_{LS}$, or at risk of reinfection at a rate $\left(1-q\right)\lambda$, where $q$ is the reduction in susceptibility to reinfection due to prior exposure to TB.

Individuals with TB disease were stratified into the three clinically and epidemiologically relevant states of smear-positive pulmonary ($I^{SP}$), smear-negative pulmonary ($I^{SN}$), and extra-pulmonary disease ($I^{EP}$), with the parameter $\alpha$ determining the fraction going into each of these disease states. Individuals with any of the pulmonary TB disease types were considered infectious, but at varying levels. Individuals in any of the TB disease states leave their state by TB-related mortality at a rate $\xi$, by spontaneous recovery at a rate $\nu$ (i.e. individuals can recover naturally without medical treatment), or by diagnosis and effective treatment at a rate $z\zeta$. Here, $\zeta$ is TB treatment rate (Equation 1.4), and $z$ is the proportion of those treated that are treated effectively and cured. Multi-drug resistant TB were modelled indirectly by reducing the treatment success proportion of those treated.

Treated individuals were stratified according to the three TB disease types: $T^{SP}$, $T^{SN}$, and $T^{EP}$, and were assumed at risk of TB reinfection at a rate $\left(1-q\right)\lambda$, TB-related mortality at a rate $\xi$, spontaneous recovery at a rate $\nu$, or successful treatment completion at a rate $\psi$. Individuals in $T^{SP}$, $T^{SN}$were considered infectious, but at varying levels.

Successfully treated individuals or those who spontaneously recover enter the recovery stage ($R$) where they remain as such unless they acquire a new TB infection at a rate $\left(1-q\right)\lambda$.

*TB transmission dynamics in presence of DM*

A fraction $f$ of individuals were assumed to enter the population as diabetics (Equation 1.2). Diabetics are at risk of DM-disease mortality at a rate $\gamma$, and follow an analogous TB natural history to that of non-diabetics. However, DM was assumed to affect TB parameters of progression into the different stages. These effects of DM on TB natural history and treatment outcomes are summarized in Table 1 of the main text (the individual effects are denoted $E_{1}$ to $E_{10}$ in Equation 1.2).

*Demography and natural mortality*

All individuals in all compartments were assumed at risk of natural mortality at a rate $\mu$. The population size (*N*) was held constant to allow the disentanglement of the epidemiological effects from the demographic effects. Accordingly, all those who die of TB-disease mortality were added back to the population as new-born susceptibles (rates of $M_{TBMorIncid}$ and $M_{DMMorIncid}$).

**1.2 Model Structure and Related Equations**

*TB transmission dynamics for the population without DM*

$\begin{matrix} \frac{dS}{dt}=\left(1-f_{DM}\right)\left(\mu N+M_{TBMorIncid}+M_{DMMorIncid}\right)-\left(\lambda+\mu\right)S \\ \frac{dL^{F}}{dt}=p\lambda S+p\left(1-q\right)\lambda L^{S}+p\left(1-q\right)\lambda\left(R+T^{SP}+T^{SN}+T^{EP}\right)-\left(\omega_{LF}+\mu\right)L^{F} \\ \frac{dL^{S}}{dt}=\left(1-p\right)\lambda S+\left(1-p\right)\left(1-q\right)\lambda\left(R+T^{SP}+T^{SN}+T^{EP}\right) -\left(p\left(1-q\right)\lambda+\omega_{LS}+\mu\right)L^{S} \\ \frac{dI^{SP}\text{ }}{dt}=\alpha^{LFtoSP}\omega_{LF}L^{F}+\alpha^{LStoSP}\omega_{LS}L^{S}-\left(\xi_{SP}+z\zeta_{SP}+\nu_{SP}+\mu\right)I^{SP} \\ \frac{dI^{SN}}{dt}\text{= }\alpha^{LFtoSN}\omega_{LF}L^{F}\text{+ }\alpha^{LStoSN}\omega_{LS}L^{S}-\left(\xi_{SN}+z\zeta_{SN}+\nu_{SN}+\mu\right)I^{SN} \\ \frac{dI^{EP}}{dt}=\alpha^{LFtoNP}\omega_{LF}L^{F}\text{+}\alpha^{LStoNP}\omega_{LS}L^{S}-\left(\xi_{NP}+z\zeta_{NP}+\nu_{NP}+\mu\right)I^{EP} \\ \frac{dT^{SP}\text{ }}{dt}=z\zeta_{SP}I^{SP}-\left(\nu_{TSP}+\psi_{TSP}+\left(1-q\right)\lambda+\xi_{TSP}+\mu\right)T^{SP} \\ \frac{dT^{SN}}{dt}\text{= }z\zeta_{SN}I^{SN}-\left(\nu_{TSN}+\psi_{TSN}+\left(1-q\right)\lambda+\xi_{TSN}+\mu\right)T^{SN} \\ \frac{dT^{EP}}{dt}=z\zeta_{NP}I^{EP}-\left(\nu_{TNP}+\psi_{TNP}+\left(1-q\right)\lambda+\xi_{TNP}+\mu\right)T^{EP} \\ \frac{dR}{dt}=\text{ }\underbrace{\nu_{SP}I^{SP}+\nu_{SN}I^{SN}+\nu_{NP}I^{EP}+\nu_{TSP}T^{SP}+\nu_{TSN}T^{SN}+\nu_{TNP}T^{EP}}_{\text{Spontaneous recovery}}+\underbrace{\psi_{TSP}T^{SP}+\psi_{TSN}T^{SN}+\psi_{TNP}T^{EP}}_{Treatment success} \\ -\left(\left(1-q\right)\lambda+\mu\right)R \end{matrix}$ **(1.1)**

*TB transmission dynamics for the population with DM*

$\begin{matrix} \frac{dS^{DM}}{dt}\text{ }=\text{ }f_{DM}\left(\mu N+M_{TBMorIncid}+M_{DMMorIncid}\right)-\left(E_{1}\lambda+\mu+\gamma\right)S^{DM} \\ \frac{dL^{F_{DM}}}{dt}\text{ }=\text{ }E_{2}pE_{1}\lambda S^{DM}+pE_{4}\left(1-q\right)\lambda L^{S_{DM}}+pE_{10}\left(1-q\right)\lambda\left(R^{DM}+T^{SP_{DM}}+T^{SN_{DM}}+T^{EP_{DM}}\right) \\ -\left(E_{3}\omega_{LF}+\mu+\gamma\right)L^{F_{DM}} \\ \frac{dL^{S_{DM}}}{dt}=\left(1-E_{2}p\right)E_{1}\lambda S^{DM}+\left(1-p\right)E_{10}\left(1-q\right)\lambda\left(R^{DM}+T^{SP_{DM}}+T^{SN_{DM}}+T^{EP_{DM}}\right) \\ -\left(pE_{4}\left(1-q\right)\lambda+E_{3}\omega_{LS}+\mu+\gamma\right)L^{S_{DM}} \end{matrix}$ **(1.2)**

$$\begin{matrix} \frac{dI^{SP_{DM}}\text{ }}{dt}=\alpha^{(LFtoSP)_{DM}}E_{3}\omega_{LF}L^{F_{DM}}+\alpha^{(LStoSP)_{DM}}E_{3}\omega_{LS}L^{S_{DM}}-\left(E_{8}z\zeta_{SP}+E_{9}\nu_{SP}+E_{7}\xi_{SP}+\mu+\gamma\right)I^{SP_{DM}} \\ \frac{dI^{SN_{DM}}}{dt}\text{= }\alpha^{(LFtoSN)_{DM}}E_{3}\omega_{LF}L^{F_{DM}}\text{+ }\alpha^{(LStoSN)_{DM}}E_{3}\omega_{LS}L^{S_{DM}}-\left(E_{8}z\zeta_{SN}+E_{9}\nu_{SN}+E_{7}\xi_{SN}+\mu+\gamma\right)I^{SN_{DM}} \\ \frac{dI^{EP_{DM}}}{dt}=\alpha^{(LFtoNP)_{DM}}E_{3}\omega_{LF}L_{a}^{F_{DM}}\text{+}\alpha^{(LStoNP)_{DM}}E_{3}\omega_{LS}L^{S_{DM}}-\left(E_{8}z\zeta_{NP}+E_{9}\nu_{NP}+E_{7}\xi_{NP}+\mu+\gamma\right)I^{EP_{DM}} \\ \frac{dT^{SP_{DM}}\text{ }}{dt}=E_{8}z\zeta_{SP}I^{SP_{DM}}-\left(E_{9}\nu_{TSP}+E_{9}\psi_{TSP}+E_{10}\left(1-q\right)\lambda+E_{7}\xi_{TSP}+\mu+\gamma\right)T^{SP_{DM}} \\ \frac{dT^{SN_{DM}}}{dt}\text{= }E_{8}z\zeta_{SN}I^{SN_{DM}}-\left(E_{9}\nu_{TSN}+E_{9}\psi_{TSN}+E_{10}\left(1-q\right)\lambda+E_{7}\xi_{TSN}+\mu+\gamma\right)T^{SN_{DM}} \\ \frac{dT^{EP_{DM}}}{dt}=E_{8}z\zeta_{NP}I^{NP_{DM}}-\left(E_{9}\nu_{TNP}+E_{9}\psi_{TNP}+E_{10}\left(1-q\right)\lambda+E_{7}\xi_{TNP}+\mu+\gamma\right)T^{EP_{DM}} \end{matrix}$$

$$\begin{matrix} \frac{dR^{DM}}{dt}=\underbrace{E_{9}\left(\nu_{SP}I^{SP_{DM}}+\nu_{SN}I^{SN_{DM}}+\nu_{NP}I^{EP_{DM}}+\nu_{TSP}T^{SP_{DM}}+\nu_{TSN}T^{SN_{DM}}+\nu_{TNP}T^{EP_{DM}}\right)}_{\text{Spontaneous recovery}} \\ +\underbrace{E_{9}\left(\psi_{TSP}T^{SP_{DM}}+\psi_{TSN}T^{SN_{DM}}+\psi_{TNP}T^{EP_{DM}}\right)}_{Treatment success}-\left(E_{10}\left(1-q\right)\lambda+\mu+\gamma\right)R^{DM} \end{matrix}$$

Here:

$M_{TBMorIncid}=\xi_{SP}I^{SP}+\xi_{SN}I^{SN}+\xi_{NP}I^{EP}+\xi_{TSP}T^{SP}+\xi_{TSN}T^{SN}+\xi_{TNP}T^{EP}$,

$M_{DMMorIncid}=\gamma(I^{SP_{DM}}+I^{SN_{DM}}+I^{EP_{DM}}+T^{SP_{DM}}+T^{SN_{DM}}+T^{EP_{DM}})$,

$$\begin{matrix} \alpha^{(LFtoSP)_{DM}}=\alpha^{(LStoSP)_{DM}}=F_{1}\alpha^{LFtoSP}, \\ \alpha^{(LFtoSN)_{DM}}=\alpha^{(LStoSN)_{DM}}=F_{2}\alpha^{LFtoSN}, \\ \alpha^{(LFtoEP)_{DM}}=\alpha^{(LStoEP)_{DM}}=\alpha^{LFtoEP},\text{ } \end{matrix}$$

$$\begin{matrix} F_{1}=\left(1+\frac{E_{5}-1}{E_{5}+1}\right)\frac{\alpha_{a}^{(LFtoSP)}+\alpha_{a}^{(LFtoSN)}}{\left[\alpha_{a}^{(LFtoSP)}\left(1+\frac{E_{5}-1}{E_{5}+1}\right)+\alpha_{a}^{(LFtoSN)}\left(1-\frac{E_{5}-1}{E_{5}+1}\right)\right]},\text{ and} \\ F_{2}=\left(1-\frac{E_{5}-1}{E_{5}+1}\right)\frac{\alpha_{a}^{(LFtoSP)}+\alpha_{a}^{(LFtoSN)}}{\left[\alpha_{a}^{(LFtoSP)}\left(1+\frac{E_{5}-1}{E_{5}+1}\right)+\alpha_{a}^{(LFtoSN)}\left(1-\frac{E_{5}-1}{E_{5}+1}\right)\right]}. \end{matrix}$$

*Force of infection*

The force of infection ($\lambda$) was determined by the probability of transmission per respiratory contact ($u$), the respiratory contact rate within the population ($\varepsilon$), and the relative infectiousness of individuals with each type of TB disease (whether untreated or treated) compared to the infectiousness of individuals with smear-positive pulmonary TB ($h$):

**(1.3)**

Mixing between individuals in the population was assumed to be random. The respiratory contact rate for a given population was obtained by fitting the model to available empirical data.

*Treatment rate*

Treatment rate in the model depended on TB disease type, and was determined using the case detection rates ($C_{DetSP}$, $C_{DetSN}$, and $C_{DetEP}$), TB-related mortality rates ($\nu_{SP}$, $\nu_{SN}$, $\nu_{EP}$), and spontaneous recovery rates ($\xi_{SP}$, $\xi_{SN}$, $\xi_{EP}$):

$\begin{matrix} \zeta_{SP}=C_{DetSP}(\nu_{SP}\text{ }+\text{ }\xi_{SP})/(1-C_{DetSP}) \\ \zeta_{SN}=C_{DetSN}(\nu_{SN}\text{ }+\text{ }\xi_{SN})/(1-C_{DetSN}) \\ \zeta_{EP}=C_{DetNP}(\nu_{NP}\text{ }+\text{ }\xi_{EP})/(1-C_{DetEP}). \end{matrix}$ **(1.4)**

The case detection rates $C_{DetSP}$, $C_{DetSN}$, and $C_{DetEP}$were obtained by fitting the model to available empirical data.

1. **Measures of the TB-DM Epidemiologic Synergy**

The epidemiologic implications of the DM effects on TB natural history and treatment outcomes were assessed using two measures of the TB-DM epidemiologic association/synergy: Hazard ratio and “true” population attributable fraction (*PAF*). In addition to the “true” *PAF*, we estimated the *PAF* using *Levin's formula* for comparison purposes.

*Hazard ratio*

The hazard ratio was defined as the ratio of TB disease incidence rate among those with DM ($I_{rate}^{DM}$) over TB disease incidence rate among those with no DM ($I_{rate}^{non-DM}$) within the same population:

$HR=\frac{I_{rate}^{DM}}{I_{rate}^{non-DM}}$. **(2.1)**

*“True” population attributable fraction*

Three “true” *PAF*s were defined as the proportion of all TB disease incidence, prevalence, or TB-related mortality in the population that could be directly (i.e. etiologically) and indirectly (i.e. effect of DM on onward TB transmission) prevented if there was no interaction between TB and DM^2^. Explicitly, it was defined as:

$PAF_{True}=\frac{D_{TB-DM}^{i}-D_{Counterfactual}^{i}}{D_{TB-DM}^{i}}$. **(2.2)**

Here, $D^{i}$ indicates the epidemiological measure of incidence, prevalence, or TB-related mortality. $D_{TB-DM}^{i}$ is the measure in a scenario where there is epidemiologic synergy between TB and DM, while $D_{Counterfactual}^{i}$ is the measure in a counter-factual scenario where there is no epidemiologic synergy between TB and DM.

*Levin's formula for the population attributable fraction*

Conceptually, the *PAF* can be understood as the proportion of TB disease in the population that could be *directly* prevented if there was no interaction between TB and DM. The classical equation for the *PAF* is provided by Levin’s formula^3^:

$PAF_{Levin}=\frac{P_{DM}(RR_{DM}-1)}{P_{DM}(RR_{DM}-1)+1}$. **(2.3)**

Here, $P_{DM}$ is the prevalence of DM in the population and $RR_{DM}$ is the relative risk for TB among DM individuals versus non-DM individuals.

1. **Data Sources**

The TB-DM interaction model was parameterized using empirical epidemiological and natural history data from multiple sources. The model’s parameter values along with their references are listed in Table S1.

**Table S1. Model assumptions in terms of parameter values.**

| **Symbol** | **Definition** | **Parameter value** | **Sources** |
| --- | --- | --- | --- |
| $\mu$ | Mortality/birth rate (per year) | $0.014$ (equivalent to life expectancy of 70 years) | 4 |
| $p$ | Proportion of TB infections entering latent-fast state | $15\%$ | 1,5 |
| $q$ | Fractional reduction in the susceptibility to TB reinfection due to prior exposure to TB | 0.65 (0.55-0.75) | 1,6 |
| $\omega_{LF}$ | Progression rate from latency to TB disease for latent-fast progressors (per year) | 0.90 (0.77-1.04) | 1 |
| $\omega_{LS}$ | Progression rate from latency to TB disease for latent-slow progressors (per year) | 0.00075 (0.00064-0.00086) | 1,6 |
| $\alpha$ | Proportions of new TB disease cases in each of the three clinical disease categories^#^ | $\begin{matrix} \alpha^{LFtoSP}=\alpha^{LStoSP}=50\% \\ \alpha^{LFtoSN}=\alpha^{LStoSN}=40\% \\ \alpha^{LFtoEP}=\alpha^{LStoEP}=10\% \end{matrix}$ | 7 |
| $\xi$ | TB disease mortality rate per TB disease category for untreated and treated cases (per year) | $\begin{matrix} \xi_{SP}=0.25\text{ }(0.213-0.288) \\ \xi_{SN}=0.1\text{ }(0.085-0.115) \\ \xi_{EP}=0.1\text{ }(0.085-0.115) \\ \xi_{TSP}=0 \\ \xi_{TSN}=0 \\ \xi_{TEP}=0 \end{matrix}$ | 1,8 |
| $z$ | Proportion of TB disease cases that are effectively treated | 84% | 1 |
| $\nu$ | Spontaneous recovery rate (per year) | $\nu_{SP}=0.1$ $\nu_{SN}=0.1$ $\nu_{EP}=0.1$ $\nu_{TSP}=0$ $\nu_{TSN}=0$ $\nu_{TEP}=0$ | 1,5,6 |
| $\psi$ | Rate of successful completion of treatment (per year) | $\begin{matrix} \psi_{TSP}=2.0\text{ }(1.70-2.30) \\ \psi_{TSN}=2.0\text{ }(1.70-2.30) \\ \psi_{TEP}=2.0\text{ }(1.70-2.30) \end{matrix}$ | 9 |
| $u$ | Transmission probability per respiratory contact | $10\%$ | 1 |
| $h$ | Relative infectiousness for each of the three disease categories and treatment categories with respect to smear-positive pulmonary disease | $\begin{matrix} h_{SP}=100\% \\ h_{SN}=25.0\% \\ h_{EP}=0.0\% \\ h_{TSP}=13.0\% \\ h_{TSN}=3.3\% \\ h_{TEP}=0.0\% \end{matrix}$ | 1,10,11 |
| **Country specific variables (India)** | | | |
| $f_{DM}$ | Proportion of the population with DM | 8.6% | 12 |
| $C$ | Case detection rate per TB disease category | $\begin{matrix} C_{DetSP}=0.82 \\ C_{DetSN}=0.65 \\ C_{DetEP}=0.65 \end{matrix}$ | Fitting parameter |
| $\varepsilon$ | Respiratory contact rate (per year) | 183.9 | Fitting parameter |
| $\gamma$ | DM-specific mortality rate (per year) | 0.001 | Fitting parameter |

^#^The three clinical categories are smear-positive pulmonary (SP), smear-negative pulmonary (SN), and extra-pulmonary (EP) tuberculosis

**ADDITIONAL FIGURES**

**Figure S1.** Proportion of tuberculosis (TB) disease incidence (**A**), prevalence (**B**), and mortality (**C**) attributed to each of the effects of diabetes mellitus (DM) on TB natural history and treatment outcomes, for all possible combinations of the five DM on TB effects that individually had the largest impact. These population attributable fraction ($PAF_{True}$) measures were estimated as the proportional reduction in the measure in a comparison between the measure in a scenario where there is TB-DM epidemiologic synergy (that is some effect for DM on TB is active), compared to a counter-factual scenario where there is no TB-DM epidemiologic synergy. Each DM on TB effect had a standardized effect size of 3.0 if the expected effect size (based on evidence) is ≥1, and (an inverse) effect size of 1/3 if the expected effect size is <1. The red dashed line in panel **A**) is the estimated *Levin's formula* population attributable fraction of 14.7%, assuming a relative risk (RR) of 3.0 for TB-disease incidence among DM versus non-DM individuals.

**
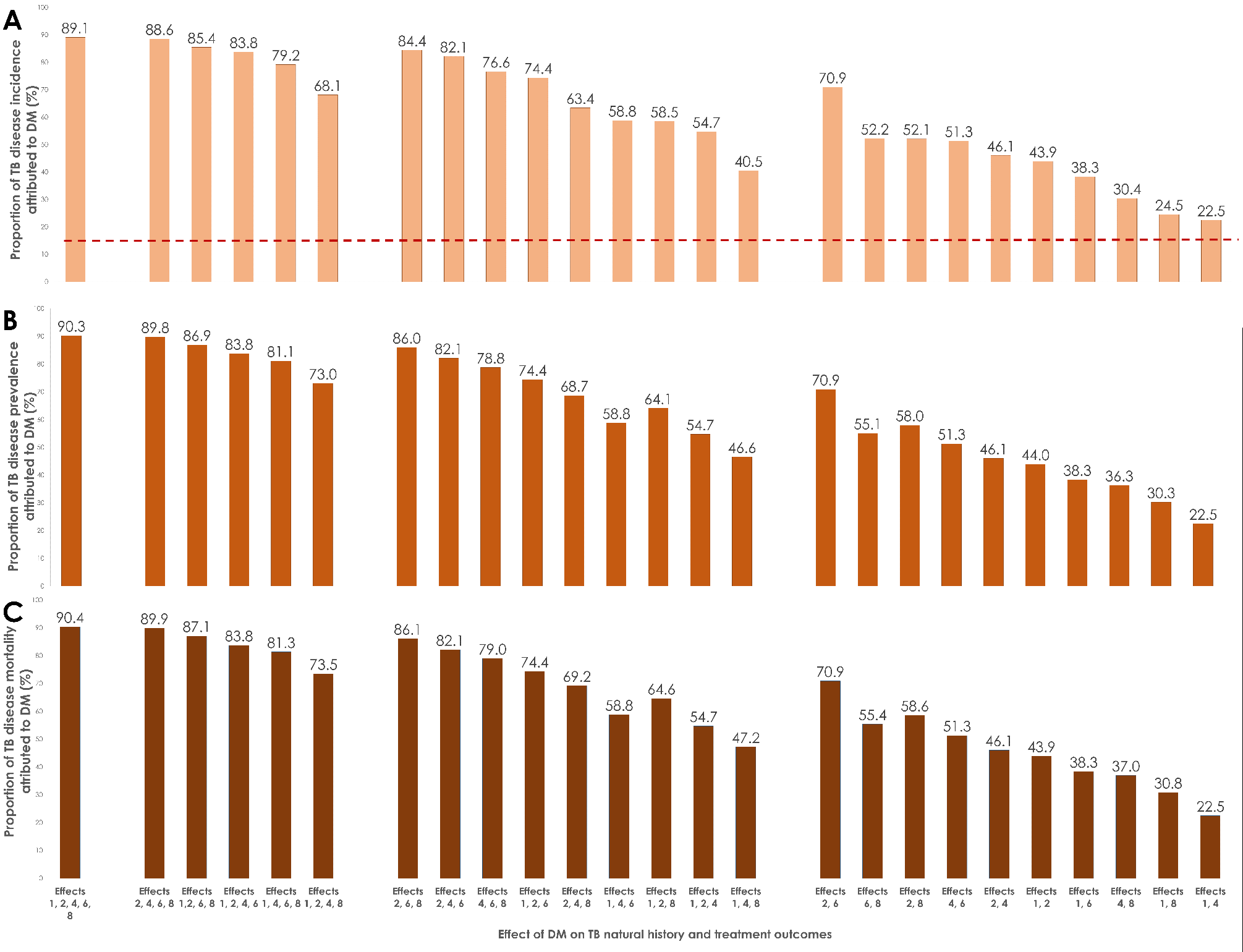
**

**Figure S2.** Epidemiological impact of all pairwise combinations of the plausible 10 effects of diabetes mellitus (DM) on tuberculosis (TB) natural history and treatment outcomes, as measured by (**A**) the incidence hazard ratio (HR) of TB disease among those with DM compared to those without DM, and by (**B**) the population attributable fraction ($PAF_{True}$). The $PAF_{True}$was estimated as the proportional reduction in incidence in a comparison between the incidence in a scenario where there is TB-DM epidemiologic synergy (that is some effect for DM on TB is active), compared to a counter-factual scenario where there is no TB-DM epidemiologic synergy. Each DM-on-TB effect had a standardized effect size of 3.0 (red dashed line in panel **A**) if the expected effect size (based on evidence) is ≥1, and (an inverse) effect size of 1/3 if the expected effect size is <1. The red dashed line in panel **B** is the estimated *Levin's formula* population attributable fraction of 14.7%, assuming a relative risk of 3.0 for TB-disease incidence among DM versus non-DM individuals.

**
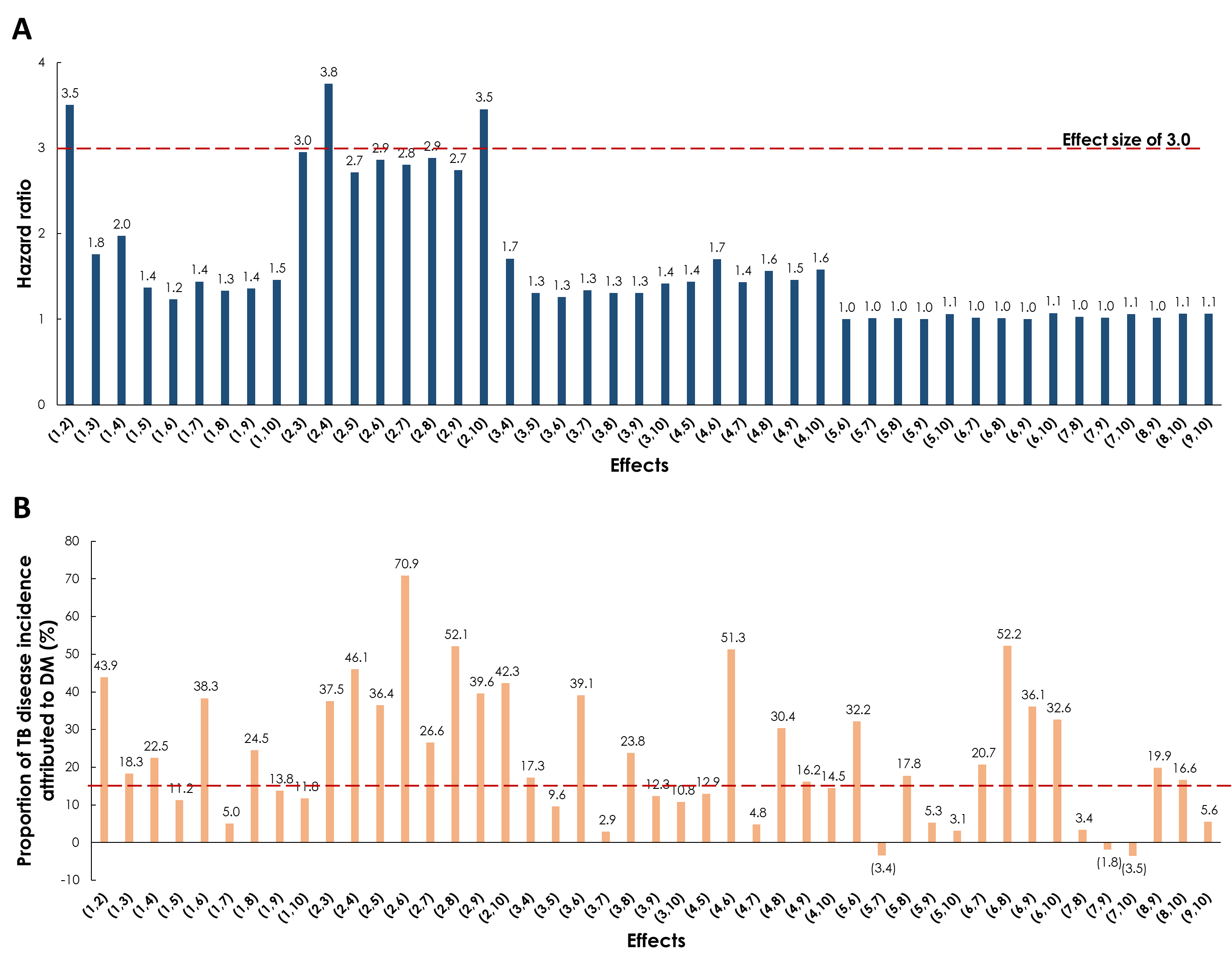
**

**Figure S3.** The incidence hazard ratio (HR) of tuberculosis disease among those with diabetes mellitus (DM) compared to those without DM of varying simultaneously the effect size (ES) of each effect in all pairwise combinations of the effects that individually had an HR >1.0 (for a standardized ES of 3.0).

**
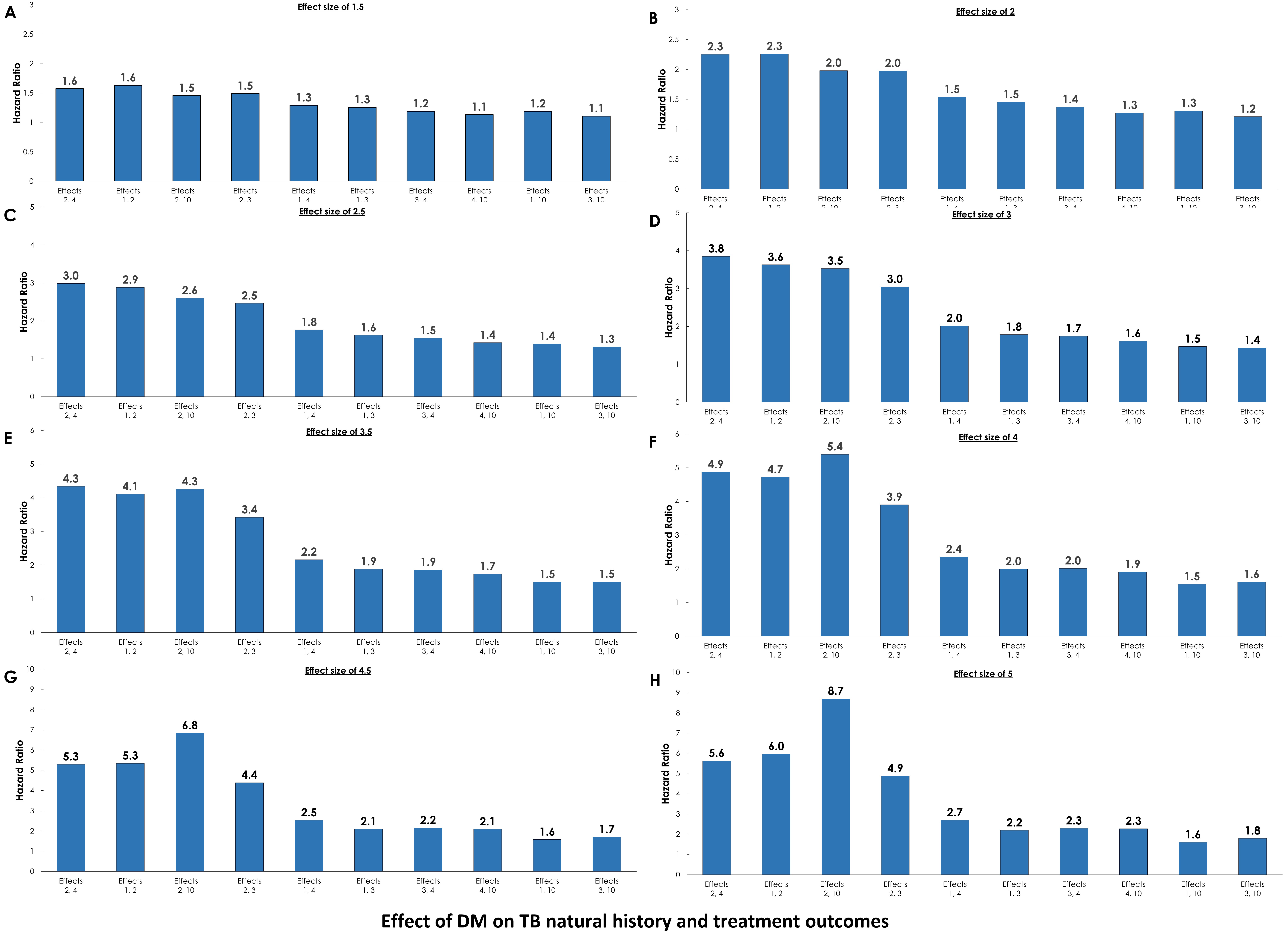
**

**REFERENCES**
